# Effectiveness of a prevention of mother-to-child HIV transmission program in Guangdong province from 2007 to 2010

**DOI:** 10.1186/1471-2458-13-591

**Published:** 2013-06-18

**Authors:** Bing Li, Qingguo Zhao, Xiaozhuang Zhang, Li Wu, Tingting Chen, Zhijiang Liang, Longchang Xu, Shouyi Yu

**Affiliations:** 1 Department of Epidemiology, School of Public Health and Tropical Medicine, Southern Medical University, Guangzhou North Road 1838, Guangzhou 510515, China; 2 Guangdong Women and Children Hospital, Xingnan Road 521, Guangzhou 511400, China

**Keywords:** HIV/AIDS, Mother to children transmission (MTCT), Anti-retroviral (ARV), Effectiveness of PMTCT

## Abstract

**Background:**

To achieve the goal of United Nations of elimination of new HIV infections, a program of prevention of mother-to-child transmission (PMTCT) was launched in Guangdong province. The objective of this study is to evaluate the effectiveness of the PMTCT program.

**Methods:**

The retrospective cross-section analysis was conducted using the data of case reported cards of HIV positive mothers and their infants from 2007 to 2010 in Guangdong province, and 108 pairs of eligible subjects were obtained. We described the data and compared the rates of MTCT by various PMTCT interventions respectively.

**Results:**

The overall rate of HIV MTCT was 13.89% (15) among 108 pairs of HIV positive mothers and their infants; 60.19% (65) of the mothers ever received ARVs, 80.56% (87) of infants born to HIV positive mothers ever received ARVs, but 16.67% (18) of the mothers and infants neither received ARVs. Among all the mothers and infants, who both received ARVs, received triple ARVs, mother received ARVs during pregnancy, and both received ARVs and formula feeding showed the lower rates of HIV MTCT, and the rates were 8.06%, 2.50%, 5.77%, and 6.67% respectively. In infants born to HIV positive mother, who received mixed feeding had a higher HIV MTCT up to 60.00%. Delivery mode might not relative to HIV MTCT.

**Conclusions:**

The interventions of PMTCT program in Guangdong could effectively reduce the rate of HIV MTCT, but the effectiveness of the PMTCT program were heavily cut down by the lower availability of the PMTCT interventions.

## Background

Mother-to-child transmission (MTCT) of human immunodeficiency virus (HIV) is the most significant route of HIV infection in children. At the United Nations 2011 High Level Meeting on AIDS, leaders committed to achieve the goal of eliminating new HIV infections among children by 2015 [1,2]. According to Estimation on China AIDS Epidemic in 2011 issued by Ministry of Health, there were an estimated 780 000 (620 000–940 000) survival people living with HIV (PLHIV), 28.6% of PLHIV were female, and 1.1% of PLHIV were infected by MTCT, there was a higher HIV prevalence among pregnant women (the highest prevalence more than 1%) in severely epidemic region [3], these data demonstrate that we face a huge challenge of Prevention of Mother-to-Child Transmission (PMTCT) in China.

However, without intervention, MTCT occurs in up to 4 of every 10 deliveries among HIV-positive women [4]. Without treatment, approximately half of the children who have acquired HIV die before two years of age, and very few survive their school years [4,5]. Due to different capability, resource and availability of health service, different region had a different result of PMTCT; the rate of MTCT could be reduced to 5-8% by integrated PMTCT program in developing country, and it could be reduced to less than 2% in some developed countries [6-8]. Guangdong is one of the higher HIV prevalence provinces in China [3], and it has initiated to implement free PMTCT program in some districts since 2003. However, the effectiveness of this program in the field are scarcely known. In order to explore this, we evaluated the effectiveness of PMTCT program with evaluating the rates of MTCT by various PMTCT interventions respectively.

## Method

### Study design

The retrospective cross-section analysis was conducted using the data of reported case cards of HIV positive mothers and their infants from 2007 to 2010 in Guangdong province of China. The case cards of HIV positive mothers were reported in five days after their HIV infected status were confirmed, and then the case cards of all infants born to HIV positive mothers were reported in five days after their HIV infected status were tested in the follow up period of 18 months. The mother’s HIV infected status was confirmed by the Western blot after her HIV antibody was positive. The infant’s HIV infected status was confirmed by the Western blot after the HIV antibody was positive at age 12 months and 18 months. If the condition was available, the infant’s HIV infected status was confirmed by HIV DAN polymerase chain reaction (PCR) tests through transported dry blood spots (DBS) at age 6 weeks to 3 months. During the follow up period, there were no death of infants, and only four infants became AIDS patients.

The targeted subjects needed to meet four requirements: 1) Born in hospital of Guangdong province from 2007 to 2010; 2) The mother’s HIV infected status was confirmed; 3) The infant born to HIV infected mother was followed up until to 18 months age and the infant’s HIV infected status was confirmed; 4) The data of ARV prophylaxis or treatment, delivery mode, and feeding were completely. We obtained 108 pairs of eligible HIV positive mothers and infants from 196 pairs of them in this article. We described the status of subjects of age, marriage status, education level, occupation, and ARV prophylaxis, and we compared the rates of MTCT respectively by various PMTCT interventions, such as ARV prophylaxis, delivery mode, and feeding mode.

### Programm description

The key interventions of the free PMTCT program included general health education on PMTCT, and provider- initiated testing and counseling (PITC) during pregnancy, safe and in hospital delivery and anti-retroviral (ARV) prophylaxis or treatment for HIV positive mothers, and formula feeding and ARV prophylaxis for infants born to HIV-positive mothers. If the HIV infected status confirmed antematally and the service condition of caesarian delivery was safe, the pregnancy women were planed to elective caesarian delivery.

With the consideration of availability of ARV drugs and existed evidences, and reference of regimen recommend by WHO since 2004, the PMTCT program of Guangdong from 2007 to 2010 used the ARV prophylaxis protocol of zidovudine (AZT) started in late pregnancy, combined with lamivudine (3TC) and single-dose nevirapine (sdNVP) during labour, and stopped in one week after delivery. And women with a CD4 count below 200 cells/mm3 were given antiretroviral treatment. Due to any unexpected reasons, if the HIV positive mothers couldn’t take the ARVs before parturient, the mothers would be offered sdNVP intrapartum as soon as possible.

### Statistic analysis

The descriptive analysis was conducted, and the mean and standard deviation were used to describe the measurement variables, and the frequency distribution was used to describe the categorical variable. The statistical significance of the comparison of proportions was determined using χ^2^ or Fisher’s exact test. The OR values and their 95% confidence intervals of the factors were estimated. The bivariate correlate was used to analyze the relationship between confirmed period of HIV infected status and whether received ARVs of mothers, and the Spearman correlation coefficient was estimated. All the statistical analyzes were done with SPSS version 13.

## Results

### Participant characteristics

Among 108 HIV positive mothers, the mean age was 27.21±5.00 years, the mean numbers of pregnancies were 3.12±1.25, the mean delivery times were 1.82±0.93; Regarding marital status, 77.78% were first marriage, 12.04% were remarriage, 2.78% were cohabitation, 7.41% were unmarried; The education degree was lower, 49.07% were junior middle school, 27.78% were senior middle school, 15.74% were primary school, and university or college education and beyond only accounted 4.63%; The majority of occupations (64.81%) were household duties or unemployed, 9.26% were workers, 9.26% were farmers, 4.63% were other occupations, and 4.63% were unknown. The detail data reference Table 1.

**Table 1:** Characteristics of pregnant HIV-infected women

**Maternal characteristics**	**n**	**%**
**Maternal age,median (range)**		
**Marital status**		
First marriage	84	77.78
Remarriage	13	12.04
Cohabitation	3	2.78
Unmarried	8	7.41
**Education**		
Primary school	17	15.74
Junior middle school	53	49.07
Senior middle school	30	27.78
College degree or above	5	4.63
Illiteracy	2	1.85
Unknown	1	0.93
**Occupation**		
Household duties or unemployed	70	64.81
Workers	10	9.26
Farmers	10	9.26
Others	13	12.04
Unknown	5	4.63
**Parity**		
Primiparity	49	45.37
Multiparity	59	54.63

Among 108 infants born to HIV positive mother, 58.3% (63) were male, and 41.7% (45) were female, and the mean birth gestation age was 38.38±1.81 weeks, and 90.74% (98) of them born at or after 37 gestation weeks.

### ARV prophylaxis

In all of 108 HIV positive mothers, only 60.19% (65) of them ever received ARVs prophylaxis from 28 weeks to one week of postpartum, but only 37.04% (40) of mothers received triple ARVs regimen.

In the 52 (48.15%, 52/108) mothers who received ARVs prophylaxis antenatally, 59.62% (31) received AZT, 26.92% (14) received sdNVP+3TC+AZT, 3.85% (2) received 3TC+AZT.

In the 60 (55.56%, 60/108) mothers who received ARVs prophylaxis intrapartum, 66.67% (40) received sdNVP+3TC+AZT, 15.00% (9) received sdNVP+AZT, 10.00% (6) received AZT, 8.33% (5) received 3TC+AZT.

In the 56 (51.85%, 56/108) mothers who received ARVs prophylaxis postnatal, 48.21% (27) were 3TC+AZT, 30.36% (17) were sdNVP+3TC+AZT, 17.86% (10) were AZT, 1.79% (1) were sdNVP+AZT, 1.79% (1) were 3TC.

There were 80.56% (87/108) of infants born to HIV positive mothers received ARVs prophylaxis, and in these 87 infants, 77.01% (67) of them received sdNVP+AZT, 10.34% (9) received AZT, 8.05% (7/87) received sdNVP, 3.45% (3) received 3TC+AZT, 1.15% (1) received 3TC+sdNVP+AZT.

Among 108 pairs of HIV positive mothers and their infants, 16.67% (18) of them neither received ARVs, 2.78% (3) of them only mothers received ARVs, 23.15% (25) of them only infants received ARVs, 57.41% (62) of them both received ARVs.

### Confirmed period of HIV infected status of mothers

As to the confirmed period of HIV infected status of 108 mothers, 5.56% (6) were before the present pregnancy, 53.70% (58) were antenatal of this pregnancy, 18.52% (20) were in the intrapartum of this pregnancy, 22.22% (24) were in the postpartum of this pregnancy. The proportion of non-received ARVs of mothers who confirmed HIV infected status before the present pregnancy, antenatal, intrapartum, and postpartum was respectively 33.33% (2/6), 8.62% (5/58), 75.00% (15/20) and 87.50% (21/24). There was a correlation between confirmed period of HIV status and whether received ARVs, and the Spearman correlation coefficient was 0.66 (T=8.97, P<0.001), which illustrated more later confirmed HIV infected status was more less possibly received ARVs of mothers.

### The mode of delivery and feeding

Among 108 pairs of mothers and infants, 22.22% (24) of infants delivered by emergency C-section, 45.37% (49) of infants delivered by vagina, and 32.41% (35) of infants delivered by elective C-section; Formula feeding was 91.67% (99), breast feeding was 3.70% (4), and mixed feeding was 4.63% (5). There were 55.56% (60) of them both mothers and infants received ARVs and formula feeding, and 24.07% (26) of them received all the four interventions including both mothers and infants received ARVs, elective C-section, and formula feeding.

### The effect of received ARVs, delivery mode, and feeding type on MTCT of HIV

The overall rate of HIV MTCT was 13.89% (15) among 108 infants born to HIV positive mothers. The data in Table 2 showed that in all the mothers and infants, who both received ARVs, received triple ARVs, mother received ARVs antenatally, and both received ARVs and formula feeding showed the lower rates of HIV MTCT, and the rates were 8.06%, 2.50%, 5.77%, and 6.67% respectively. In infants born to HIV positive mother, who received mixed feeding had a higher HIV MTCT up to 60.00%. Delivery mode might not relative to HIV MTCT. MTCT of HIV was eliminated among the 26 subjects who received all the four interventions including both mothers and infants received ARVs, elective C-section, and formula feeding.

**Table 2:** The relationships between ARVs, delivery mode or feeding type and MTCT of HIV (n=108)

**Interventions of PMTCT**	**Infants’ HIV test results**		**Total**	**χ**^**2**^**value**	***P*-value**	**OR(95% CI)**
	**Positive N (%)**	**Negative N (%)**				
Who received ARVs				Fisher’s Exact Test	0.07	
Neither	5(27.78)	13(72.22)	18			4.38(1.11, 17.40)
Mother only or infant only	5(17.86)	23(82.14)	28			2.48(0.65, 9.38)
Both	5(8.06)	57(91.94)	62			1.0
Mother’s ARVs regimen				6.90	0.032	
None any ARVs	9(20.93)	34(79.07)	43			10.32(1.24, 85.71)
Non-triple ARVs	5(20.00)	20(80.00)	25			9.75(1.07, 89.20)
Triple ARVs	1(2.50)	39(97.50)	40			1.0
Mother received ARVs antenatally				5.53	0.02	4.45(1.18, 16.83)
Yse	3(6.12)	49(94.23)	52			
No	12(27.27)	44(78.57)	56			
Mother and infant both received ARVs and formula feeding				6.51	0.039	
None of three	2(33.33)	4(66.67)	6			7.0(0.97,50.57)
One or two of three	9(21.43)	33(78.57)	42			3.82(1.09,13.38)
All of three	4(6.67)	56(93.33)	60			1.0
Mother and infant both received ARVs and formula feeding and elective C-section				Fisher’s Exact Test	0.01	
None of four	2(33.33)	4(66.67)	6			1.50(0.85,2.64)
One to three of four	13(17.11)	63(82.89)	76			1.21(1.09,1.34)
All of four	0(0.00)	26(100.00)	26			1.0
Feeding type				Fisher’s Exact Test	0.04	
Mixed feeding	3(60.00)	2(40.00)	5			10.88(1.65,71.86)
Breast feeding	0(0.00)	4(100.00)	4			0.88(0.82,0.95)
Formula feeding	12(12.12)	87(87.88)	99			1.0
Delivery mode				Fisher’s Exact Test	0.49	
Emergency C-section	3(12.50)	21(87.50)	24			0.63(0.16, 2.60)
Vagina delivery	3(8.57)	32(91.43)	35			0.42(0.10, 1.67)
Elective C-section	9(18.37)	40(81.63)	49			1.0
Total	15(13.89)	93(86.11)	108			

The rate of HIV MTCT of infants whose mothers ever received ARVs and non-received ARVs was 9.23% (6/65) and 20.93% (9/43) respectively (p>0.05), and that of infants who received and non-received ARVs was 10.34% (9/87) and 28.57% (6/21) respectively (p>0.05).

## Discussion

The study showed that the overall rate of HIV MTCT was 13.89% in Guangdong province from 2007 to 2010 after PMTCT interventions were implemented, and it was a MTCT rate of HIV similar to resource-limited region [8-10], and did not achieve the expectant goal of reducing the rate of HIV MTCT down to less than 5%. One of the main reasons of un-achieved the goal was the lower availability of the PMTCT interventions. The data of this article showed a lower and out-timely availability of ARVs of HIV positive mothers and their infants. Only 60.19% of HIV positive mothers received ARVs, and only 48.15% of them received ARVs during pregnancy, and merely 60.19% of them received triple ARVs regimen, and 80.56% of infants born to them received ARVs, and the formula feeding rate was 91.67%, and only 55.56% of mothers and their infants both received ARVs and formula feeding.

Consideration the conditions and surroundings of the PMTCT program, maybe many causes resulted in the lower availability of the PMTCT interventions. Such as, not enough HIV test ability of primary health service institutions, not enough training on PMTCT for primary medical personnel, HIV positive mothers initially participated antenatal care lately and even until to nearly delivery, following up difficulty for flowing population and worrying about discrimination, pregnant women do not know about the PMTCT interventions, and the ARVs for infants need to be imported and can’t be provided timely. A lot of literatures reported that many influencing factors can result in missing opportunities for prevention of mother-to-child transmission of HIV among HIV positive mothers and their infants, such as people’s knowledge about PMTCT, the antenatal service system and HIV test ability of facilities, following up mode [11-17]. The data of this study showed that 18.52% of HIV positive mothers confirmed HIV infected status during delivery, and 22.22% of them confirmed HIV infected status postnatal, and that resulted in lower availability of ARVs.

Although there was no significant difference of overall HIV-MTCT rate between mothers or infants received ARVs and those non-received ARVs, the data showed that mothers and their infants neither received ARVs compared to those both received ARVs, mothers received non-triple ARVs compared to those received triple ARVs, and mothers non-received ARVs during pregnancy compared to those received ARVs during pregnancy, can increase the HIV-MTCT rate, and the OR value was 4.38 (95% CI: 1.11-17.40), 9.75 (95% CI: 1.07-89.20) and 4.32 (95% CI: 1.09-17.15) respectively. If HIV positive mothers receive ARVs following the referred regimen, the HIV-MTCT rate could be decreased to 2.5%; and if HIV positive mothers received ARVs during pregnancy, the HIV-MTCT rate could be decreased to 5.77%, and the effects of PMTCT were similar to that in developed countries [8,10,18,19]. The data illustrated that HIV positive mothers and their infants received ARVs timely and routinely can help to decrease the HIV MTCT rate significantly, and the helping was limited if they received ARVs out-time or un-routinely.

The data of this study showed that mixed feeding increased the opportunity of HIV-MTCT compared to formula feeding (OR=10.88, 95% CI: 1.65-71.86), and decreased the opportunity of HIV-MTCT compared to breast feeding (OR=0.88, 95% CI: 0.82-0.95)). The study of Gerardo et al. revealed that universal ARV therapy to HIV-infected pregnant women is able to reduce mother-to-child transmission to less than 5%, although breastfeeding can increase the rate of HIV transmission, formula feeding produces malnutrition during the first month of life, increases mortality compared to breastfeeding [20]. The results of whether breastfeeding increased the rate of HIV MTCT was different, but only 4 infants received breastfeeding in our data, it needed more samples to be further analyzed.

Siobhan et al. reported that there was no association between mode of delivery and HIV MTCT, which was consistent with our findings [21]. But we found that when HIV positive mothers and their infants received ARVs and formula feeding, and plus elective C-section could further reduce HIV MTCT. Mothers and their infants both received ARVs and formula feeding could reduce HIV MTCT down to 6.67% (4/60), but HIV MTCT was eliminated in 26 mothers and their infants who received all the interventions including received ARVs, elective C-section, and formula feeding.

The review of Tudor et al. described that integrated PMTCT services could reduce MTCT effectively [22]. The study of Ngozi et al. showed that holistic but cost effective preventive interventions help in reducing the rate of mother-to-child transmission of HIV even in economically-developing settings [23]. Turan et al. found that PMTCT of HIV and antenatal care integration in pregnancy might improve the implementation and effectiveness of PMTCT in rural area [24]. All of above mentioned illustrated that it was important to strengthen the implementation of PMTCT program to increase the availability of interventions in Guangdong province for achieving the goal of elimination of new infections among children by 2015, which was similar to the problems of developing country [25].

A number of clinical trials have showed that MTCT can now be reduced to less than 2 percent from a possible 25–30 percent without any intervention [17]. With the cumulation of evidence of PMTCT, the recommendation protocol of PMTCT is kept on update. The finding of this study denominated that the protocol of PMTCT program in Guangdong from 2007 to 2010 could effectively reduce the rate of MTCT, but that effectiveness were heavily cut down by the lower availability of integrated PMTCT interventions. Although recently the evidence showed that the breastfeeding could be use for PMTCT in many developing countries, but the formula-feeding was more benefit to PMTCT in the setting of Guangdong province.

In addition, the data of this article showed that there was no significant decrease of HIV MTCT rate in the funded PMTCT regions compared to non-funded PMTCT regions (11.29% vs. 17.39%) after the PMTCT program was launched in Guangdong. And the loss to follow-up among infants was the main obstacle of effective evaluation of PMTCT program, there were 196 pairs of HIV positive mothers and their infants were reported form 2007 to 2010 in Guangdong, only 55.1% (108/196) of them were eligible. All of the excluded objects were due to loss to follow-up the HIV infectious status of infants, which mostly because of population flowing and the parent was afraid of impact to the life of their child after the HIV infectious status was exposed. According to the information of mothers’ reported cards, in 88 subjects who lost to follow up, the mean age of mother was 28.78±5.28 years, which was higher than the mean age of targeted subject (t=2.13, p=0.03); But the distributions of marital status, education level, occupation, and the rate of ARV prophylaxis were no significant difference respectively between lost follow up mothers and targeted mothers (p>0.05). And 67.05% (59/88) of mothers and 79.55% (70/88) of their infants ever received ARVs among 88 subjects who lost to follow up. According to these data, it was suppose that the estimation of MTCT rates in this study was acceptable.

## Conclusions

At present, the data showed that the overall rate of HIV MTCT was 13.89% in Guangdong province from 2007 to 2010 after PMTCT interventions were implemented, which did not achieve the expectant goal of reducing the rate of HIV MTCT down to less than 5%. But in the HIV positive mothers and their infants who could receive the interventions recommended by PMTCT program, the rate of HIV MTCT might be reduced down to below 5.0%. Therefore, the interventions of PMTCT program in Guangdong could effectively reduce the rate of HIV MTCT, but the effectiveness of the PMTCT program were heavily cut down by the lower availability of the PMTCT interventions.

### Ethical consideration

Ethical approval was obtained from Guangdong Women and Children Hospital Ethics Committee.

## Competing interests

The authors declare that they have no competing interests.

## Authors’ contributions

LB performed the statistical analysis and drafted the manscript; LJ, XC and CT collected the data. ZG, ZZ and YY conceived the study; YY and WL revised the mansucript. All authors read and approved the final manuscript.

## Pre-publication history

The pre-publication history for this paper can be accessed here:

http://www.biomedcentral.com/1471-2458/13/591/prepub
